# The Metallurgy of Platinized Gold Cast Clasps

**Published:** 1922-08

**Authors:** M. Elisberg

**Affiliations:** New York. N. Y.


### THE METALLURGY OF PLATINIZED GOLD CAST CLASPS

BY M. ELISBERG, NEW YORK. N. Y.

It has often been stated, that the success of cast clasps depends upon the metal used and also upon the alloys used in the metal. This is partly true. The success of the cast clasps depends upon the basic metal, but it does not depend upon the alloys. The metal depends upon the alloys. Any metal that can be successfully beaded with an engraving tool can be successfully cast, and if that metal can retain its spring it is just the thing for clasps. The success of the clasp also depends upon the method of melting, and of course the right preparation of the investment material.

Nickel as an alloy, used in platinized gold, has been often unjustly abused, mostly by people who have failed to produce a successful metal with its use. It has a high fusing point, does not burn out when used with gold, and has high coloring and polishing qualities. The only objection possible to its use as an alloy is the color of its oxide, which is a dark purple, almost black. However, this has been successfully counteracted by the introduction of zinc whose white oxide forces this nickel oxide into a combination grey oxide, an oxide easily washed off and which is not injurious—in fact having healing qualities. Nickel does not cause crystallization, which is one of the causes of brittleness in metals, unless used in quantities over fifteen per cent.

Palladium, as an alloy, is used to whiten gold. Platinum as an alloy raises the fusing point of gold and if used in sufficient quantity will whiten but will also crystallize. To give resilience or springiness to gold, copper is usually added. Copper in this combination causes a black oxide which is easily pickled, *i. e.*, eaten up readily by acid. A chemist of high standing claims that any form of copper oxide is poisonous. These metals are used also in making platinized gold. This form of platinized gold does not take as high a polish as the one containing nickel.

In melting metals care should be taken in the use of the same, and flux should not be spared. The metal should first be moistened and then well sprinkled with a suitable casting flux, such as animal charcoal, then brought to a molten state on a charcoal block, in which a groove has been carved, with the tip of the blue flame from the blowpipe. While at red heat and in solid form the resulting button should be transferred to the crucible of the investment, enough flux added to completely cover the button and the tip of the blue flame again applied to bring the metal to a molten state; as soon as this is accomplished the blowpipe should be moved back, according to the fusing point of the metal, so that as cool a flame as possible is used to keep it in a molten state; the flame should then be applied all around the edge of the molten metal, then to the center of the top, and then cast. All this should be done as quickly as possible. The reason for the result of most brittle clasps is the burning out of alloys caused by a more than necessary use of the flame, or the use of too hot a flame after the metal is in a molten state. Another cause is the throwing apart of the alloys caused by a concentrated flame.

The use of yellow gold in dentistry is on the wane, due to the esthetic superiority of platinized golds and palladium golds.—*Dental Digest.*

